# Statistical models for the analysis of skewed healthcare cost data: a simulation study

**DOI:** 10.1186/s13561-015-0045-7

**Published:** 2015-05-27

**Authors:** Amal Saki Malehi, Fatemeh Pourmotahari, Kambiz Ahmadi Angali

**Affiliations:** Department of Biostatistics and Epidemiology, School of Public Health, Ahvaz Jundishapur University of Medical Sciences, Ahvaz, Iran

**Keywords:** Skewed data, Generalized linear models (GLMs), Cox proportional hazard regression, Ordinary least squares (OLS) model, Transformation, Healthcare cost, Monte Carlo simulation

## Abstract

Skewed data is the main issue in statistical models in healthcare costs. Data transformation is a conventional method to decrease skewness, but there are some disadvantages. Some recent studies have employed generalized linear models (GLMs) and Cox proportional hazard regression as alternative estimators.

The aim of this study was to investigate how well these alternative estimators perform in terms of bias and precision when the data are skewed. The primary outcome was an estimation of population means of healthcare costs and the secondary outcome was the impact of a covariate on healthcare cost. Alternative estimators, such as ordinary least squares (OLS) for Ln(y) or Log(y), Gamma, Weibull and Cox proportional hazard regression models, were compared using Monte Carlo simulation under different situations, which were generated from skewed distributions.

We found that there was not one best model across all generated conditions. However, GLMs, especially the Gamma regression model, behaved well in the estimation of population means of healthcare costs. The results showed that the Cox proportional hazard model exhibited a poor estimation of population means of healthcare costs and the β_1_ even under proportional hazard data. Approximately results are consistent by increasing the sample size. However, increasing the sample size could improve the performance of the OLS-based model.

## Background

Statistical models are often used in many healthcare economics and policy studies. The main issues in such studies are the estimation of mean population healthcare costs and finding the best relationship between costs and covariates through regression modeling [1]. However, these cannot be implemented by simple statistical models as the healthcare costs data have specific characterizations [2]. Healthcare costs data demonstrate the substantial positive skewness and are sometimes characterized by the use of large resources with zero cost [3]. These specifications of data impose a number of difficulties in using standard statistical analysis, such as implementing linear regression causes unreliable results [2].

Two-part models based on mixture models are performed when excess zeroes are present in data [3]. Further, logarithmic (or other) transformations are commonly used to decrease the skewness and drive them close to normal distribution, in order to implement linear regression models. The logarithmic transformation with ordinary least squares (OLS) regression is a very common approach in applied economics. However, it also presents several drawbacks. One of these drawbacks is that the predictions are not robust enough to detect the heteroscedasticity in the transformed scale [1,4]. The general consensus is that estimating the mean cost using a logarithmic regression model leads to biased estimation [2,4-6].

An alternative approach is using nonlinear regression models, of which exponential conditional mean (ECM) models in generalized linear models (GLMs) are examples [7]. Generally, GLMs extend the linear modeling framework to allow response variables that are not normally distributed. In healthcare studies, generalized linear modeling through log-link function avoids the weakness and problems of OLS regression. In addition, the Cox proportional hazards model has been a controversial issue for healthcare data modeling. It has been used as a special flexible model for skewed healthcare data in many studies [8,9].

In recent years, extensive research efforts have been done to propose suitable regression methods for the analysis of skewed healthcare data [1,3,10,11]. Many studies also set out a clear framework for comparing these methods from a variety of aspects [5,6,12,13]. Moreover, a few have provided prominent reviews of the statistical methods for analyzing healthcare data [2,7].

However, there is no comparative study that investigates the different methods using different sample sizes. This paper was conducted to compare the proposed statistical models in the available literature using different sample sizes. We specifically focused on comparing proposed statistical models for positive skewed healthcare costs, but not zero mass problems. It was developed based on a Monte Carlo simulation to find appropriate methods to get the unbiased and precise estimates of the mean costs. This aspect is particularly important in the literature [5,13]. Furthermore, in this paper, the coefficient estimations of covariates are also evaluated in our simulations using different sample sizes.

## Methods

Let *y*_*i*_ denote healthcare expenditures for person *i*, and *x*_*i*_ denote the set of covariates, including the intercept. We estimated the following models.

### Ordinary least square based on log transformation

It is common to use linear regression models for the log of costs in healthcare expenditures. Logarithmic transformation is most commonly used to decrease skewness and to make the distribution more symmetric and closer to normality. The log regression model is as follows:

$$ \ln \left({y}_i\right)={x}_i\beta +{\varepsilon}_i $$

Where it was assumed that *E*(*xε*) = 0 and *E*(*ε*) = 0, since predicting costs on the original scale is primary objective so:

$$ {y}_i= \exp \left({x}_i\beta +{\varepsilon}_i\right) $$

$$ E\left(\left.{y}_i\right|{x}_i\right)=E\left( \exp \left({\varepsilon}_i\right)\Big|{x}_i\right) \exp \left({x}_i\beta \right) $$

If the error term is $N\left(0,{\sigma}_{\upvarepsilon}^2\right)$ distribution, it is a log-normal case, and then:

$$ E\left(\left.{y}_i\right|{x}_i\right)= \exp \left({x}_i\beta +0.5{\sigma}_{\upvarepsilon}^2\right) $$

However, if the error term is not normally distributed, but is homoscedastic, then the smearing estimator is applied.

### Generalized linear models

GLMs are a broad class of statistical models for relating non-normal dependent variables to linear combinations of predictor variables. An invertible link function (g (.)) converts the expectation of the response variable, *E (Y*_*i*_ *)*, to the linear predictor:

$$ g\left(E\left({y}_i\right)\right)=g\left({\mu}_i\right)={x}_i\beta $$

The ECM model is a special type of GLM with log-link function, and can be viewed as a nonlinear regression model:

$$ E\left(\left.{y}_i\right|{x}_i\right)= \exp \left({x}_i\beta \right) $$

Weibull and Gamma regression models are assumed as two special types of ECM model; *β* values were estimated here using quasi-maximum likelihood estimation. The exponential distribution was considered to be a special case of the Weibull and Gamma regression models when the shape parameter was equal to 1.

### Cox proportional hazard model

The Cox proportional hazard model is based on hazard and survival functions, instead of ECM or direct estimation of *E (y|x)*. It is a semi-parametric model because it does not specify the baseline hazard function:

$$ h\left(\left.{y}_i\right|{x}_i\right)={h}_0(y) \exp \left({x}_i\beta \right) $$

Where *h*_0_(*y*) is the baseline hazard, estimated using the Breslow method. The main issue in this model, which should be considered, is the proportional hazard assumption. This means that the hazard ratio of two individuals is independent of time [14]. Note that the interpretation of the estimated coefficients in this model is based on hazard ratio rather than the covariate effect on the mean.

### Comparing model performance

The interested estimations are evaluated as follows:

Two statistics were calculated to evaluate the quality of cost predictions using above mentioned models. The first was the mean prediction error (MPE), which measures the bias and predictive accuracy, and the second was the mean absolute prediction error (MAPE):

$$ MPE=\frac{1}{n}{\displaystyle \sum_{i=1}^n\left({y}_i-{\widehat{y}}_i\right)} $$

$$ MAPE=\frac{1}{n}{\displaystyle \sum_{i=1}^n\left|{y}_i-{\widehat{y}}_i\kern0.1em \right|} $$

Actually, MPE indicates how the mean of predicted healthcare expenditures from a particular model compares with the mean of healthcare costs. Models with lower values of MPE have smaller biases than models with higher values. However, MAPE indicates how values of individual predicted healthcare expenditures from a particular model compare with values of actual healthcare expenditures in the sample [6].

Mean square of error (MSE) and 95% confidence interval of the estimate of β_1_ coefficient were calculated to evaluate the accuracy and precision of the estimated parameter. A more precise estimator should be closer to the true value. A Goodness of fit test provided by Hosmer-Lemeshow test and the Akaike information criteria (AIC) used as an aid to choosing between competing models. Lower values of the AIC indicate the preferred model criterion were also used to evaluate. The mean of the residuals across deciles of x was also plotted in order to assess a systematic bias in the predictions.

### Simulation study

To compare the performance of the alternative models, a Monte Carlo simulation was used to show how each estimator behaves under different conditions of skewness that are common in healthcare expenditure studies.

To determine the effect of the level of skewness on the estimated outcome, some skewed probability density function (pdf), such as log-normal, Gamma and Weibull distribution, was used as a data-generating mechanism. To assess how the sample size affects the estimations, 10,000 times batch samples (n = 25, 50, 100, 500 and 1,000) were examined by comparing all models mentioned in the previous section. All generated data were standardized according to Basu *et al*., in which *β*_0_ was considered as intercept, estimated assuming *E(y) = 1*.

### Log-normal data generation

The true model assumed is as follows:

$$ \ln (y)={\beta}_0+{\beta}_1+\varepsilon $$

Where *x* is uniform (0, 1), *ε* ∼ *N*(0, *σ* ^2^), in which *σ* ^2^ = 0.5, 1.0, 1.5, and *β*_1_ = 1 were used. *β*_0_ was estimated based on *E(y) =1*:

$$ E\left(y\Big|\;x\right)= \exp \left({\beta}_0+{\beta}_1x+0.5{\sigma}^2\right) $$

The skewness of log-normal models is an increasing function of the variance as follows:

$$ \left( \exp \left({\sigma}^2\right)+2\right){\left( exp\left({\sigma}^2\right)-1\right)}^{0.5} $$

We considered *σ* ^2^ = 0.5, 1, 1.5 and 2.

### Gamma data generation

The pdf of Gamma distribution is:

$$ f(y)=\frac{1}{\varGamma \left(\alpha \right){b}^{\alpha }}{y}^{\alpha -1}{e}^{-y/b} $$

Where *b* = exp(*β*_0_ + *β*_1_ *x*) and *α* are the scale and shape parameters, respectively. The mean is equal to *αb* and the skewness is a decreasing function of the shape parameter, as follows:

$$ \frac{2}{\sqrt{\alpha }} $$

Where *x* is uniform (0, 1), *β*_1_ = 1 and *β*_0_ was estimated so that *E(y) = 1.* Also, we used the assumption that *α* = 0.5, 1, 2 and 4 in the data generating process.

### Weibull data generation

Weibull data generation is considered as a function of the data-generating mechanism, which has proportional hazard properties. It was used to generate proportional hazard data, since the Cox proportional hazards model is based on this assumption:

$$ f(y)=\frac{\alpha }{b}{\left(\frac{y}{b}\right)}^{\alpha +1}{e}^{{\left(-y/b\right)}^{\alpha }} $$

Where *b* = exp(*β*_0_ + *β*_1_ *x*) and *α* are the scale and shape parameters, respectively. The mean is equal to $b\varGamma \left(1+\frac{1}{\alpha}\right)$ and the skewness is also a decreasing function of the shape parameter, as follows:

$$ {b}^3\varGamma \left(1+\frac{3}{\alpha}\right)-3\varGamma \left(1+\frac{1}{\alpha}\right)\varGamma \left(1+\frac{2}{\alpha}\right)+2{\left(\varGamma \left(1+\frac{1}{\alpha}\right)\right)}^3 $$

Shape parameter was considered as 0.5, 1 and 5 in this scenario. The proportional hazards assumption was evaluated in all of the simulations.

## Results

Mean, standard deviation, skewness and kurtosis for the *y* in various data-generating mechanisms are presented in Table 1. Based on this result, the log-normal and Weibull models provided greater skewness than the Gamma model. It should be noted that the skewness of data from the log-normal and Gamma models increased monotonically as the sample size increased.

**Table 1:** **Simple statistics of y**

		**Mean**	**Std. Dev.**	**Coefficient of skewness**	**Coefficient of kurtosis**
	Log normal σ^2^=0.5	1.000	0.827	1.615	5.890
	Log normal σ^2^=1	1.000	1.200	2.070	7.684
	Log normal σ^2^=1.5	1.000	1.524	2.368	9.017
	Log normal σ^2^=2	1.000	1.813	2.585	10.057
n=25	Gamma α=0.5	1.000	1.402	1.962	6.885
	Gamma α =1	1.000	1.022	1.544	5.400
	Gamma α =2	1.000	0.760	1.247	4.565
	Gamma α =4	1.000	0.576	1.040	4.051
	Wiebull α=0.5	1.000	1.939	2.592	9.902
	Wiebull α =1	1.000	1.028	1.565	5.488
	Wiebull α =5	1.000	0.363	0.668	3.131
	Log normal σ^2^=0.5	1.000	0.841	1.992	8.305
	Log normal σ^2^=1	1.000	1.251	2.669	12.101
	Log normal σ^2^=1.5	1.000	1.626	3.132	15.086
	Log normal σ^2^=2	1.000	2.060	3.476	17.481
n=50	Gamma α=0.5	1.000	1.433	2.350	9.558
	Gamma α =1	1.000	1.049	1.824	7.064
	Gamma α =2	1.000	0.769	1.459	5.691
	Gamma α =4	1.000	0.579	1.192	4.788
	Wiebull α=0.5	1.000	2.073	3.334	16.015
	Wiebull α =1	1.000	1.047	1.846	7.182
	Wiebull α =5	1.000	0.361	0.666	3.234
	Log normal σ^2^=0.5	1.000	0.868	2.339	11.213
	Log normal σ^2^=1	1.000	1.307	3.293	18.377
	Log normal σ^2^=1.5	1.000	1.736	3.983	24.446
	Log normal σ^2^=2	1.000	2.159	4.512	29.521
n=100	Gamma α=0.5	1.000	1.466	2.681	12.454
	Gamma α =1	1.000	1.071	2.064	8.819
	Gamma α =2	1.000	0.781	1.615	6.665
	Gamma α =4	1.000	0.588	1.292	5.328
	Wiebull α=0.5	1.000	2.178	4.095	24.487
	Wiebull α =1	1.000	1.074	2.074	8.861
	Wiebull α =5	1.000	0.370	0.626	3.054
	Log normal σ^2^=0.5	1.000	0.888	2.892	18.063
	Log normal σ^2^=1	1.000	1.364	4.667	40.650
	Log normal σ^2^=1.5	1.000	1.880	6.206	65.574
	Log normal σ^2^=2	1.000	2.420	7.508	89.605
n=500	Gamma α=0.5	1.000	1.492	3.106	17.456
	Gamma α =1	1.000	1.076	2.320	11.267
	Gamma α =2	1.000	0.789	1.764	7.819
	Gamma α =4	1.000	0.594	1.369	5.826
	Wiebull α=0.5	1.000	2.293	5.650	51.600
	Wiebull α =1	1.000	1.077	2.317	11.208
	Wiebull α =5	1.000	0.374	0.573	2.884
	Log normal σ^2^=0.5	1.000	0.882	3.030	20.532
	Log normal σ^2^=1	1.000	1.387	5.167	53.191
	Log normal σ^2^=1.5	1.000	1.914	7.197	94.542
	Log normal σ^2^=2	1.000	2.492	9.016	137.859
n=1000	Gamma α=0.5	1.000	1.495	3.192	18.720
	Gamma α =1	1.000	1.078	2.367	11.805
	Gamma α =2	1.000	0.791	1.786	8.018
	Gamma α =4	1.000	0.597	1.381	5.909
	Wiebull α=0.5	1.000	2.313	6.179	65.070
	Wiebull α =1	1.000	1.078	2.360	11.684
	Wiebull α =5	1.000	0.373	0.575	2.872

The results in Tables 2, 3, 4, 5 and 6 were based on 10,000 times batch replication, in sample sizes of 25, 50, 100, 500 and 1,000, respectively. These tables show the results of the estimates of population means and β_1_ for each model under the various data-generating processes. Minimum deviance (MPE) and absolute deviance (MAPE) of predicting the value of the response variable (health-care costs) considered as adequacy of methods.

**Table 2:** **Alternative estimator results for log-normal, gamma and weibull distributions for n=25**

**Data**	**Estimator**	**MPE**	**MAPE**	**MSE(β)**	**95% CI**		**AIC**	**Prob. H.Lsignif**
					**Lower**	**upper**		
Log normal σ^2^=0.5	OLS for Ln(y)	-0.13903	0.58026	0.28579	0.798	1.214	56.527	0.0484
	Gamma	-0.00070	0.53623	0.24738	0.765	1.221	43.796	0.0453
	Weibull	-0.11815	0.57319	0.25534	0.742	1.236	45.032	0.0493
	Cox	-1.45570	3.85240	6.77976	-1.823	-1.089	114.191	0.0522
Log normal σ^2^=1	OLS for Ln(y)	-0.14087	0.80071	0.57158	0.715	1.303	73.856	0.0467
	Gamma	-0.00259	0.74803	0.47688	0.637	1.332	49.636	0.0432
	Weibull	-0.02790	0.75177	0.51067	0.635	1.333	49.889	0.0451
	Cox	-1.02151	3.67692	4.79504	-1.374	-0.670	115.543	0.0581
Log normal σ^2^=1.5	OLS for Ln(y)	-0.14266	0.96247	0.85736	0.651	1.371	83.992	0.0481
	Gamma	-0.00667	0.90470	0.69826	0.523	1.427	48.094	0.0440
	Weibull	0.08439	0.85470	0.76599	0.553	1.407	47.547	0.0442
	Cox	-0.83058	3.61682	4.04647	-1.179	-0.483	116.007	0.0544
Log normal σ^2^=2	OLS for Ln(y)	-0.14384	1.08909	1.14315	0.597	1.429	91.184	0.0485
	Gamma	-0.01478	1.03115	0.91562	0.420	1.514	43.316	0.0429
	Weibull	0.19665	0.91580	1.02132	0.484	1.470	42.107	0.0414
	Cox	-0.71755	3.58418	3.63860	-1.06	-0.373	116.245	0.0536
Gamma α=0.5	OLS for Ln(y)	-0.30508	1.10870	4.184	0.327	1.646	112.098	0.1269
	Gamma	-0.00608	0.93533	1.831	0.514	1.405	40.684	0.0468
	Weibull	0.22314	0.86661	2.132	0.509	1.426	41.359	0.0455
	Cox	-0.70630	3.61984	3.532	-1.054	-0.359	116.236	0.0534
Gamma α =1	OLS for Ln(y)	-0.16364	0.76291	1.424	0.626	1.380	85.253	0.0727
	Gamma	-0.00141	0.70474	0.854	0.687	1.289	51.104	0.0470
	Weibull	-0.01889	0.70780	0.858	0.686	1.290	51.072	0.0481
	Cox	1.07902	3.67304	4.794	-1.412	-0.714	115.454	0.0546
Gamma α =2	OLS for Ln(y)	-0.14447	0.55706	0.567	0.779	1.240	62.351	0.0545
	Gamma	-0.00064	0.51805	0.422	0.760	1.203	45.250	0.0461
	Weibull	-0.11319	0. 54472	0.406	0.773	1.202	45.302	0.0485
	Cox	1.52397	3.95791	6.794	-1.887	-1.161	113.989	0.0583
Gamma α =4	OLS for Ln(y)	-0.13872	0.40613	0.248	0.847	1.166	42.011	0.0479
	Gamma	-0.00020	0.37338	0.208	0.851	1.150	32.861	0.0431
	Weibull	-0.12969	0.40265	0.200	0.840	1.151	33.311	0.0471
	Cox	-2.18196	4.31535	10.402	-2.572	-1.792	111.303	0.0486
Wiebull α=0.5	OLS for Ln(y)	-0.34517	1.36816	3.73002	0.251	1.761	119.821	0.1253
	Gamma	-0.02216	1.15326	1.73985	0.296	1.600	22.472	0.0448
	Weibull	0.43461	0.95799	2.23442	0.349	1.581	22.094	0.0408
	Cox	-0.51486	3.57624	2.98777	-0.948	-0.082	116.549	0.0531
Wiebull α =1	OLS for Ln(y)	-0.16807	0.76539	0.93251	0.626	1.380	85.164	0.0702
	Gamma	-0.00210	0.70482	0.56343	0.676	1.290	51.009	0.0492
	Weibull	-0.01845	0.70757	0.55860	0.675	1.291	50.971	0.0502
	Cox	-1.04789	3.75803	4.92479	-1.489	-0.607	115.443	0.0526
Wiebull α =5	OLS for Ln(y)	-0.13691	0.20584	0.03730	0.926	1.076	4.692	0.0526
	Gamma	-0.00006	0.17590	0.03153	0.930	1.068	0.040	0.0412
	Weibull	-0.08524	0.18546	0.02234	0.935	1.059	-2.112	0.0470
	Cox	-5.24388	7.34860	40.76941	-5.785	-4.703	96.674	0.0526

**Table 3:** **Alternative estimator results for log-normal, gamma and weibull distributions for n=50**

**Data**	**Estimator**	**MPE**	**MAPE**	**MSE(β)**	**95% CI**		**AIC**	**Prob. H.Lsignif**
					**Lower**	**upper**		
Log normal σ^2^=0.5	OLS for Ln(y)	-0.06472	0.56174	0.14414	0.901	1.109	110.247	0.0403
	Gamma	-0.00024	0.54325	0.12915	0.880	1.112	84.882	0.0377
	Weibull	-0.11401	0.58013	0.13512	0.865	1.119	87.987	0.0416
	Cox	-1.37774	3.67486	5.99725	-1.550	-1.206	292.456	0.0507
Log normal σ^2^=1	OLS for Ln(y)	-0.06560	0.77896	0.28826	0.861	1.153	144.905	0.0375
	Gamma	-0.00084	0.75579	0.24681	0.809	1.169	97.178	0.0332
	Weibull	-0.01498	0.75773	0.27025	0.809	1.169	97.694	0.0344
	Cox	-0.96876	3.53907	4.20450	-1.135	-0.803	295.126	0.0536
Log normal σ^2^=1.5	OLS for Ln(y)	-0.06646	0.93700	0.43240	0.830	1.188	165.178	0.0346
	Gamma	-0.00204	0.91116	0.35880	0.743	1.219	94.667	0.0309
	Weibull	0.10499	0.85852	0.40537	0.766	1.206	93.005	0.0298
	Cox	-0.78847	3.49213	3.52210	-0.952	-0.624	296.053	0.0556
Log normal σ^2^=2	OLS for Ln(y)	-0.06989	1.10461	0.57653	0.803	1.217	179.5625	0.0347
	Gamma	-0.00465	1.07701	0.46796	0.680	1.266	89.735	0.0307
	Weibull	0.23242	0.95573	0.54049	0.730	1.238	86.227	0.0281
	Cox	-0.68152	3.46853	3.14852	-0.846	-0.520	296.522	0.0504
Gamma α=0.5	OLS for Ln(y)	-0.13425	1.01591	2.105	0.675	1.334	222.881	0.1086
	Gamma	-0.00197	0.94922	0.891	0.772	1.208	77.941	0.0351
	Weibull	0.24545	0.87554	1.055	0.770	1.219	79.168	0.0346
	Cox	-0.70741	3.51983	3.211	-0.871	-0.544	296.415	0.0531
Gamma α =1	OLS for Ln(y)	-0.07705	0.47464	0.702	0.813	1.190	168.791	0.0608
	Gamma	-0.00047	0.28527	0.426	0.847	1.144	100.154	0.0388
	Weibull	-0.00937	0.28340	0.428	0.847	1.145	100.134	0.0389
	Cox	1.03789	0.33563	4.397	-1.198	-0.871	294.821	0.0531
Gamma α =2	OLS for Ln(y)	-0.06760	0.54581	0.278	0.886	1.125	122.363	0.0498
	Gamma	-0.00026	0.53020	0.212	0.896	1.106	87.850	0.0438
	Weibull	-0.11172	0.55696	0.201	0.893	1.106	88.214	0.0470
	Cox	1.47746	3.80179	6.397	-1.648	-1.307	291.826	0.0504
Gamma α =4	OLS for Ln(y)	-0.06486	0.39403	0.123	0.927	1.087	81.482	0.0456
	Gamma	-0.00003	0.38221	0.106	0.928	1.079	63.053	0.0424
	Weibull	-0.13114	0.41234	0.103	0.923	1.080	64.471	0.0471
	Cox	-2.09719	4.10274	9.736	-2.282	-1.912	286.445	0.0496
Wiebull α=0.5	OLS for Ln(y)	-0.15405	1.25405	1.89494	0.638	1.396	237.978	0.1004
	Gamma	-0.00678	1.16471	0.84376	0.652	1.304	43.032	0.0352
	Weibull	0.47033	0.96587	1.14195	0.690	1.296	41.454	0.0333
	Cox	-0.50825	3.47052	2.60197	-0.754	-0.264	297.097	0.0504
Wiebull α =1	OLS for Ln(y)	-0.07916	0.74709	0.47373	0.819	1.199	168.664	0.0625
	Gamma	-0.00076	0.72112	0.28681	0.845	1.147	99.360	0.0416
	Weibull	-0.00859	0.72241	0.28548	0.844	1.148	99.339	0.0418
	Cox	-1.02239	3.63137	4.43438	-1.272	-0.776	294.819	0.0521
Wiebull α =5	OLS for Ln(y)	-0.06425	0.18584	0.01895	0.964	1.040	7.720	0.051
	Gamma	-0.00003	0.18068	0.01658	0.967	1.035	-1.858	0.0452
	Weibull	-0.08750	0.19046	0.01142	0.969	1.029	-6.490	0.0534
	Cox	-5.11234	6.96179	38.13497	-5.360	-4.864	256.001	0.0493

**Table 4:** **Alternative estimator results for log-normal, gamma and weibull distributions for n=100**

**Data**	**Estimator**	**MPE**	**MPAE**	**MSE(β)**	**95% CI**		**AIC**	**Prob. H.Lsignif**
					**Lower**	**upper**		
Log normal σ^2^=0.5	OLS for Ln(y)	-0.03144	0.56088	0.06312	0.953	1.049	217.5766	0.0391
	Gamma	-0.00007	0.55234	0.05761	0.942	1.052	168.199	0.0361
	Weibull	-0.11282	0.58936	0.06098	0.935	1.057	175.260	0.0417
	Cox	-1.34295	3.32199	5.63414	-1.423	-1.263	716.154	0.0481
Log normal σ^2^=1	OLS for Ln(y)	-0.03161	0.77499	0.12623	0.933	1.069	286.891	0.0365
	Gamma	-0.00020	0.76419	0.10963	0.907	1.081	192.904	0.0333
	Weibull	-0.00812	0.76533	0.12196	0.908	1.080	193.907	0.0330
	Cox	-0.94387	3.19872	3.91711	-1.020	-0.868	722.133	0.0479
Log normal σ^2^=1.5	OLS for Ln(y)	-0.03195	0.93383	0.18935	0.917	1.085	327.438	0.0335
	Gamma	-0.00038	0.92175	0.15884	0.873	1.107	189.222	0.0300
	Weibull	0.11681	0.86782	0.18295	0.887	1.099	185.001	0.0294
	Cox	-0.76851	3.15738	3.26405	-0.844	-0.694	724.207	0.0531
Log normal σ^2^=2	OLS for Ln(y)	-0.03217	1.05939	0.25247	0.904	1.098	356.206	0.0320
	Gamma	-0.00068	1.04672	0.20674	0.840	1.132	172.665	0.0283
	Weibull	0.23968	0.92933	0.24393	0.869	1.113	163.925	0.0276
	Cox	-0.66436	3.13647	2.90548	-0.738	-0.590	725.262	0.0544
Gamma α=0.5	OLS for Ln(y)	-0.06210	0.98793	0.924	0.842	1.149	444.474	0.1015
	Gamma	-0.00071	0.95946	0.382	0.899	1.099	151.970	0.0366
	Weibull	0.25749	0.88296	0.456	0.896	1.102	154.259	0.0380
	Cox	0.69973	3.18874	2.997	-0.700	-0.626	724.990	0.050
Gamma α =1	OLS for Ln(y)	-0.03843	0.74577	0.307	0.915	1.093	335.557	0.0569
	Gamma	-0.00026	0.73384	0.185	0.934	1.072	196.691	0.0391
	Weibull	-0.00460	0.73458	0.185	0.934	1.072	196.682	0.0395
	Cox	-1.02065	3.27855	4.182	-1.095	-0.947	721.285	0.0518
Gamma α =2	OLS for Ln(y)	-0.03271	0.54277	0.120	0.946	1.057	242.168	0.0504
	Gamma	-0.00011	0.53579	0.092	0.950	1.494	171.847	0.0434
	Weibull	-0.11069	0.56268	0.087	0.949	1.049	172.908	0.0471
	Cox	-1.44678	3.44580	6.080	-1.525	-1.369	714.645	0.0503
Gamma α =4	OLS for Ln(y)	-0.03138	0.39126	0.053	0.966	1.040	160.228	0.0436
	Gamma	-0.00001	0.38627	0.046	0.967	1.037	122.262	0.0403
	Weibull	-0.13163	0.41676	0.044	0.964	1.038	125.708	0.0515
	Cox	-2.05432	3.72857	9.359	-2.138	-1.970	702.730	0.0506
Wiebull α=0.5	OLS for Ln(y)	-0.07169	1.20997	0.82955	0.830	1.186	473.993	0.0833
	Gamma	-0.00180	1.16992	0.36191	0.839	1.145	83.622	0.032
	Weibull	0.48656	0.96925	0.50264	0.856	1.138	79.302	0.0345
	Cox	-0.49779	3.13454	2.38376	-0.668	-0.330	726.558	0.0485
Wiebull α =1	OLS for Ln(y)	-0.03853	0.74709	0.20739	0.915	1.093	335.3635	0.0574
	Gamma	-0.00025	0.73522	0.12587	0.928	1.068	196.7417	0.0399
	Weibull	-0.00400	0.73582	0.12566	0.928	1.068	196.7316	0.0397
	Cox	-1.00326	3.28425	4.16180	-1.176	-0.834	721.3257	0.0505
Wiebull α =5	OLS for Ln(y)	-0.03115	0.18335	0.00829	0.983	1.019	13.476	0.0480
	Gamma	-0.00001	0.18277	0.00738	0.984	1.016	-7.0357	0.0437
	Weibull	-0.08850	0.19266	0.00503	0.986	1.014	-16.598	0.0639
	Cox	-5.04559	6.57363	36.88155	-5.160	-4.932	636.392	0.0472

**Table 5:** **Alternative estimator results for log-normal, gamma and weibull distributions for n=500**

**Data**	**Estimator**	**MPE**	**MPAE**	**MSE(β)**	**95% CI**		**AIC**	**Prob. H.Lsignif**
					**Lower**	**upper**		
Log normal σ^2^=0.5	OLS for Ln(y)	-0.00617	0.55823	0.01166	0.991	1.011	1075.552	0.0438
	Gamma	-0.000002	0.55662	0.01079	0.989	1.011	830.756	0.0405
	Weibull	-0.11093	0.59335	0.01155	0.987	1.011	870.429	0.0538
	Cox	-1.31086	3.10119	5.36566	-1.326	-1.296	5157.713	0.0490
Log normal σ^2^=1	OLS for Ln(y)	-0.00625	0.76743	0.02331	0.987	1.015	1422.125	0.0444
	Gamma	-0.00002	0.76539	0.02041	0.981	1.017	953.996	0.0380
	Weibull	-0.00211	0.76577	0.02309	0.982	1.016	958.951	0.0382
	Cox	-0.92086	3.01376	3.71427	-0.935	-0.907	5189.630	0.0543
Log normal σ^2^=1.5	OLS for Ln(y)	-0.00646	0.92875	0.03497	0.985	1.019	1624.858	0.0406
	Gamma	-0.00004	0.92652	0.02935	0.974	1.022	945.716	0.0338
	Weibull	0.12644	0.87192	0.03464	0.978	1.020	919.644	0.0351
	Cox	-0.74999	2.98739	3.08671	-0.764	-0.736	5200.723	0.0474
Log normal σ^2^=2	OLS for Ln(y)	-0.00665	1.05164	0.04662	0.983	1.021	1768.699	0.0407
	Gamma	-0.00006	1.04944	0.03788	0.966	1.028	867.320	0.0316
	Weibull	0.25187	0.93223	0.04619	0.974	1.024	813.451	0.0371
	Cox	-0.64857	2.97510	2.74186	-0.663	-0.635	5206.363	0.0500
Gamma α=0.5	OLS for Ln(y)	-0.01173	0.97145	0.170	0.966	1.026	2218.380	0.0814
	Gamma	-0.00010	0.96635	0.069	0.981	1.019	745.079	0.0395
	Weibull	0.26621	0.88808	0.082	0.979	1.018	756.009	0.0613
	Cox	-0.69386	3.04111	2.896	-0.999	-0.388	5204.358	0.050
Gamma α =1	OLS for Ln(y)	-0.00739	0.73625	0.056	0.984	1.018	1669.842	0.0582
	Gamma	-0.00001	0.73405	0.034	0.987	1.014	960.724	0.0431
	Weibull	-0.00095	0.73423	0.034	0.987	1.014	960.723	0.0438
	Cox	-1.00444	3.10634	4.035	-1.019	-0.990	5184.427	0.0468
Gamma α =2	OLS for Ln(y)	-0.00643	0.54150	0.022	0.999	1.013	1202.164	0.0452
	Gamma	-0.00002	0.54021	0.017	0.992	1.011	844.867	0.0403
	Weibull	-0.10982	0.56708	0.016	0.992	1.011	851.287	0.0546
	Cox	-1.42736	3.23880	5.909	-1.442	-1.413	5148.590	0.0461
Gamma α =4	OLS for Ln(y)	-0.00606	0.39091	0.010	0.993	1.007	792.221	0.0443
	Gamma	0.000004	0.39006	0.008	0.993	1.007	598.026	0.0416
	Weibull	-0.13200	0.42060	0.008	0.993	1.007	617.434	0.1017
	Cox	-2.01502	3.48489	9.092	-2.031	-1.999	5086.403	0.0486
Wiebull α=0.5	OLS for Ln(y)	-0.01379	1.18150	0.15321	0.962	1.032	2362.321	0.0606
	Gamma	-0.00012	1.17416	0.06475	0.965	1.025	411.304	0.0338
	Weibull	0.49762	0.97207	0.09307	0.969	1.025	384.861	0.0693
	Cox	-0.49022	2.99166	2.25145	-0.563	-0.421	5213.082	0.0495
Wiebull α =1	OLS for Ln(y)	-0.00741	0.73714	0.03830	0.980	1.016	1669.173	0.0530
	Gamma	-0.00002	0.73494	0.02327	0.984	1.012	961.400	0.0421
	Weibull	-0.00082	0.73506	0.02326	0.984	1.012	961.376	0.0418
	Cox	-0.99154	3.11589	4.00036	-1.066	-0.922	5184.367	0.0473
Wiebull α =5	OLS for Ln(y)	-0.00605	0.18346	0.00153	0.996	1.004	59.7355	0.0453
	Gamma	-0.000003	0.18362	0.00138	0.997	1.003	-51.535	0.0447
	Weibull	-0.08896	0.19356	0.00093	0.997	1.003	-101.476	0.2244
	Cox	-5.00813	6.36391	36.15827	-5.029	-4.987	4737.774	0.0530

**Table 6:** **Alternative estimator results for log-normal, gamma and weibull distributions for n=1000**

**Data**	**Estimator**	**MPE**	**MPAE**	**MSE(β)**	**95% CI**		**AIC**	**Prob. H.Lsignif**
					**Lower**	**upper**		
Log normal σ^2^=0.5	OLS for Ln(y)	-0.00311	0.55282	0.00586	0.996	1.006	2147.649	0.0488
	Gamma	-0.00001	0.55202	0.00543	0.994	1.006	1642.073	0.0436
	Weibull	-0.10959	0.58828	0.00583	0.994	1.006	1722.864	0.0701
	Cox	-1.30433	3.10889	5.32271	-1.312	-1.296	11694.099	0.0467
Log normal σ^2^=1	OLS for Ln(y)	-0.00326	0.77307	0.01172	0.995	1.009	2840.796	0.0488
	Gamma	-0.00001	0.77202	0.01028	0.990	1.008	1924.378	0.0419
	Weibull	-0.00120	0.77225	0.01166	0.990	1.008	1934.411	0.0417
	Cox	-0.91650	3.02844	3.68525	-0.923	-0.909	11757.613	0.0467
Log normal σ^2^=1.5	OLS for Ln(y)	-0.00339	0.92803	0.01759	0.994	1.010	3246.261	0.0477
	Gamma	-0.00002	0.92689	0.01477	0.986	1.010	1893.638	0.0393
	Weibull	0.12788	0.87225	0.01749	0.988	1.010	1839.946	0.0433
	Cox	-0.74664	3.00457	3.06286	-0.754	-0.740	11779.664	0.0479
Log normal σ^2^=2	OLS for Ln(y)	-0.00351	1.05067	0.02344	0.993	1.013	3533.943	0.0463
	Gamma	-0.00002	1.04957	0.01904	0.981	1.013	1738.981	0.0354
	Weibull	0.25118	0.92331	0.02331	0.987	1.011	1607.688	0.0480
	Cox	-0.64582	2.99362	2.72102	-0.653	-0.639	11790.872	0.0543
Gamma α=0.5	OLS for Ln(y)	-0.00551	0.96948	0.085	0.978	1.007	4435.972	0.0845
	Gamma	-0.00001	0.96709	0.034	0.989	1.008	1487.309	0.0417
	Weibull	0.26721	0.88856	0.041	0.989	1.007	1508.951	0.0931
	Cox	-0.69278	3.06113	2.881	-0.700	-0.686	11786.66	0.0505
Gamma α =1	OLS for Ln(y)	-0.00374	0.73620	0.028	0.992	1.009	3337.268	0.0540
	Gamma	-0.000001	0.73511	0.017	0.993	1.006	1919.125	0.0420
	Weibull	-0.00042	0.73519	0.017	0.993	1.006	1919.131	0.0417
	Cox	-1.00124	3.1238	4.015	-1.008	-0.994	11747.09	0.0529
Gamma α =2	OLS for Ln(y)	-0.00318	0.54246	0.011	0.995	1.006	2401.279	0.0481
	Gamma	-0.00001	0.54183	0.009	0.995	1.005	1691.20	0.0447
	Weibull	-0.10998	0.56877	0.008	0.995	1.005	1704.418	0.0785
	Cox	-1.42245	3.24810	5.882	-1.430	-1.415	11675.63	0.0533
Gamma α =4	OLS for Ln(y)	-0.00305	0.39286	0.005	0.996	1.003	1581.076	0.0455
	Gamma	-0.000004	0.39244	0.004	0.997	1.003	1203.85	0.0435
	Weibull	-0.13273	0.42318	0.004	0.997	1.004	1243.481	0.2093
	Cox	-2.00825	3. 48492	9.047	-2.016	-2.000	11551.56	0.0518
Wiebull α=0.5	OLS for Ln(y)	-0.00654	1.17692	0.07707	0.978	1.014	4722.98	0.0643
	Gamma	-0.00004	1.17347	0.03245	0.980	1.012	819.453	0.0378
	Weibull	0.49853	0.97136	0.04682	0.983	1.011	765.204	0.1416
	Cox	-0.48930	3.01307	2.23645	-0.543	-0.439	11804.08	0.0492
Wiebull α =1	OLS for Ln(y)	-0.00361	0.73627	0.01926	0.989	1.007	3336.686	0.0560
	Gamma	-0.00001	0.73520	0.01171	0.991	1.006	1919.109	0.0426
	Weibull	-0.00042	0.73527	0.01170	0.991	1.005	1919.06	0.0432
	Cox	-0.99001	3.13384	3.98134	-1.044	-0.940	11746.65	0.0509
Wiebull α =5	OLS for Ln(y)	-0.00301	0.18367	0.00077	0.998	1.002	117.810	0.0397
	Gamma	-0.000001	0.18377	0.00069	0.998	1.002	-105.433	0.0393
	Weibull	-0.08904	0.19371	0.00047	0.998	1.002	-205.982	0.6238
	Cox	-5.00343	6.35876	36.0715	-5.014	-4.992	10855.17	0.0485

**Figure 1:**
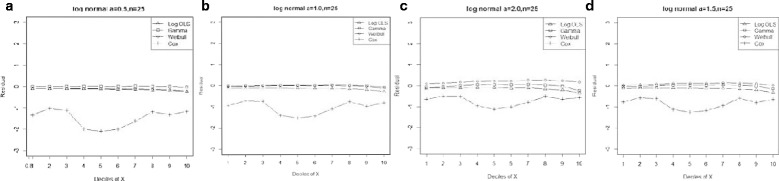
**Mean residual from different estimators across deciles of ‘X’ for log-normal data (n=25) with variance a: 0.5, b: 1.0, c: 1.5, d: 2.0.**

Generally, entire models exhibited lower MPE by declining skewness and increasing sample size. However, the Gamma regression model had the smallest biases across all data-generating processes. Moreover, our results indicated that its ability to predict the expenditures in a small sample size was as good as for large sample sizes. Furthermore, OLS for Ln(y) and Weibull regression models showed a lower bias than the Cox proportional hazard model, even in proportional hazard data-generating process (Figure 1).

**Figure 2:**
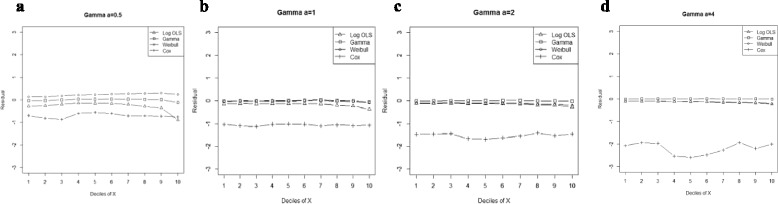
**Mean residual from different estimators across deciles of ‘X’ for Gamma data (n=25) with shape parameter a: 0.5, b: 1, c: 2, d: 4.**

**Figure 3:**
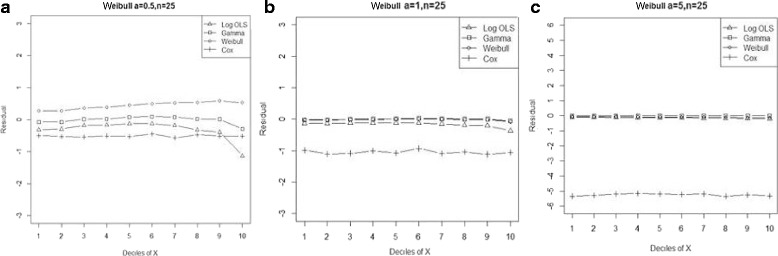
**Mean residual from different estimators across deciles of ‘X’ for Weibull data (n=25) with shape parameter a: 0.5, b: 1, c: 5.**

**Figure 4:**
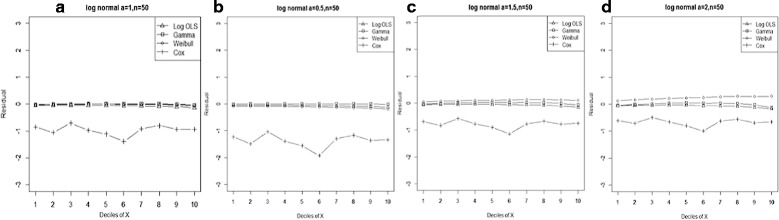
**Mean residual from different estimators across deciles of ‘X’ for log-normal data (n=50) with variance a: 0.5, b: 1.0, c: 1.5, d: 2.0.**

**Figure 5:**
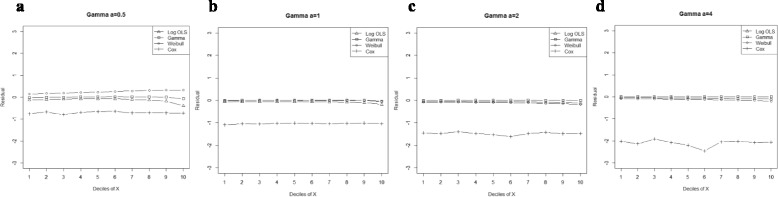
**Mean residual from different estimators across deciles of ‘X’ for Gamma data (n=50) with shape parameter a: 0.5, b: 1, c: 2, d: 4.**

**Figure 6:**
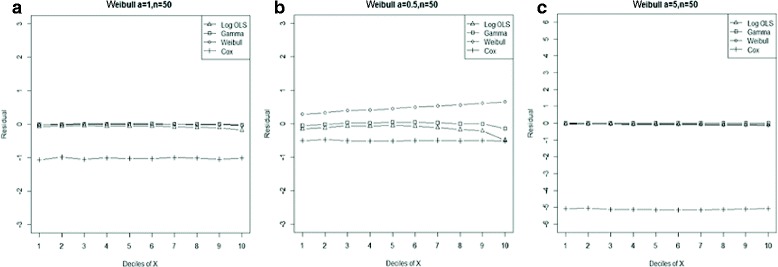
**Mean residual from different estimators across deciles of ‘X’ for Weibull data (n=50) with shape parameter a: 0.5, b: 1, c: 5.**

**Figure 7:**
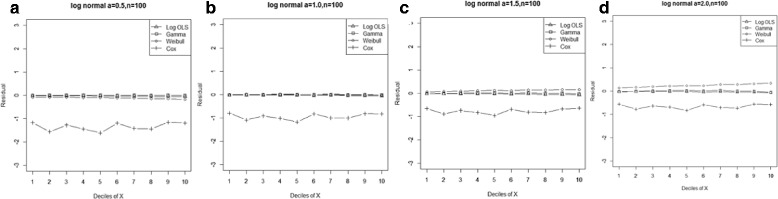
**Mean residual from different estimators across deciles of ‘X’ for log-normal data (n=100) with variance a: 0.5, b: 1.0, c: 1.5, d: 2.0.**

**Figure 8:**
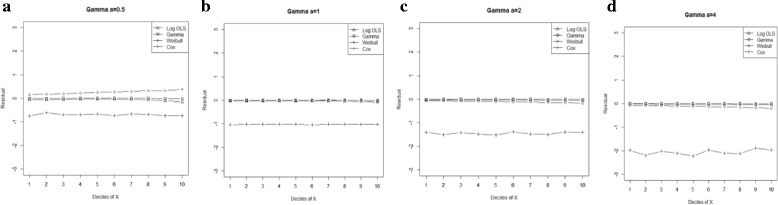
**Mean residual from different estimators across deciles of ‘X’ for Gamma data (n=100) with shape parameter a: 0.5, b: 1, c: 2, d: 4.**

**Figure 9:**
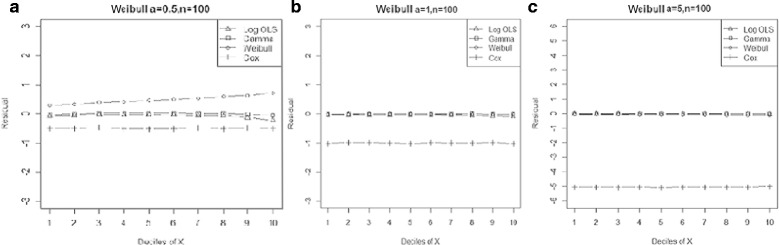
**Mean residual from different estimators across deciles of ‘X’ for Weibull data (n=100) with shape parameter a: 0.5, b: 1, c: 5.**

**Figure 10:**
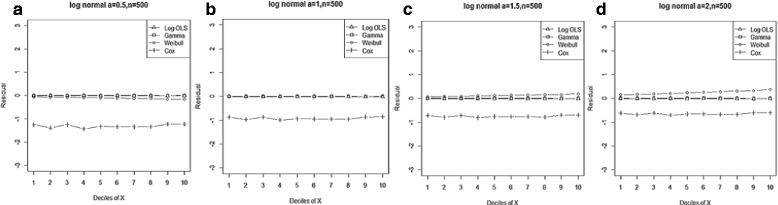
**Mean residual from different estimators across deciles of ‘X’ for log-normal data (n=500) with variance a: 0.5, b: 1.0, c: 1.5, d: 2.0.**

**Figure 11:**
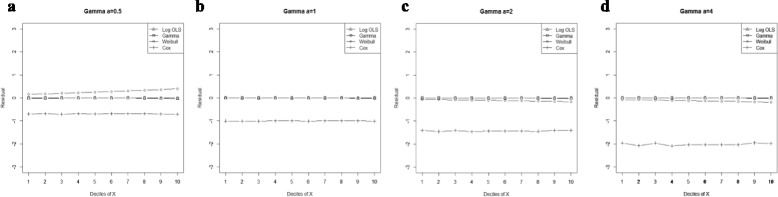
**Mean residual from different estimators across deciles of ‘X’ for Gamma data (n=500) with shape parameter a: 0.5, b: 1, c: 2, d: 4.**

**Figure 12:**
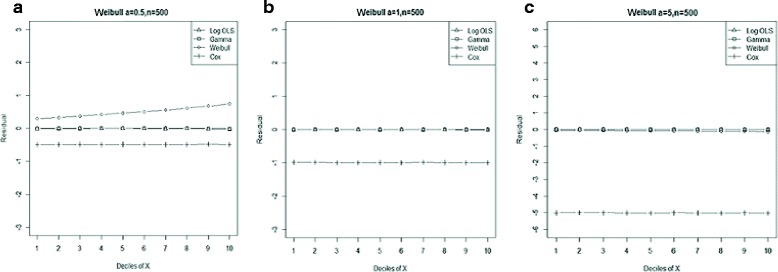
**Mean residual from different estimators across deciles of ‘X’ for Weibull data (n=500) with shape parameter a: 0.5, b: 1, c: 5.**

**Figure 13:**
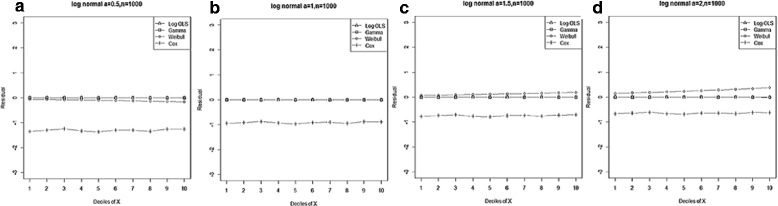
**Mean residual from different estimators across deciles of ‘X’ for log-normal data (n=1000) with variance a: 0.5, b: 1.0, c: 1.5, d: 2.0.**

**Figure 14:**
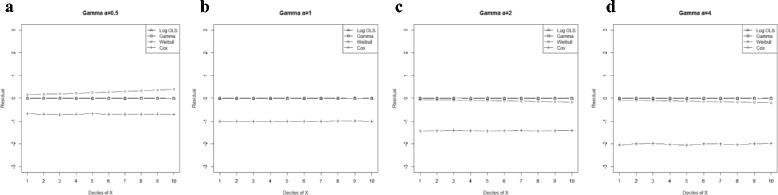
**Mean residual from different estimators across deciles of ‘X’ for Gamma data (n=1000) with shape parameter a: 0.5, b: 1, c: 2, d: 4.**

**Figure 15:**
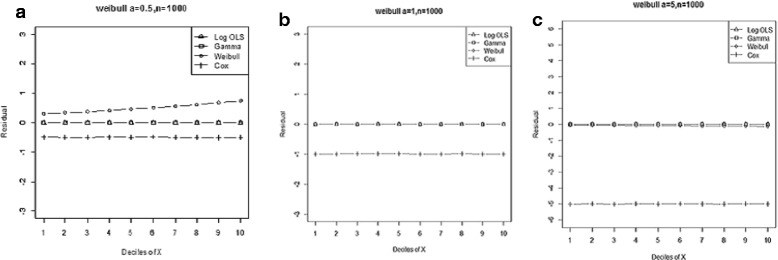
**Mean residual from different estimators across deciles of ‘X’ for Weibull data (n=1000) with shape parameter a: 0.5, b: 1, c: 5.**

In addition, evaluating MAPE as an accuracy measure showed that Gamma and Weibull regression models have almost equal MAPE values. In many conditions, such as the log-normal model with σ^2^ = 1.5, 2, the Gamma model with shape equal to 0.5 (monotonically declining pdf) and the Weibull model with shape equal to 0.5 (linearly increasing hazard), as higher skewness data-generating mechanisms, the MAPE from Weibull regression model was always lower than Gamma regression model.

Interestingly, as the sample size increased, the MAPE of OLS for Ln(y) became very similar to that of the Gamma regression model. However, the MAPE of all models had an insignificant upward trend as the sample size increased.

Since there was also a concern about consistency and precision in the estimates of β_1_ coefficients, MSE and 95% simulation intervals were investigated. All three regression Gamma and Weibull and OLS for Ln(y) models provided approximately similar MSEs of β_1_ as data generated using log normal. However, the Gamma regression model showed minimum MSE values. We also found that MSE decreased by reducing skewness and increasing sample size. For the Weibull-generated data, Gamma and Weibull regression models exhibited similar and minimum values of MSE. Under all data-generating mechanisms, 95% simulation intervals were closer to true values in all three regression models. Surprisingly, the Cox proportional hazard model revealed maximum MSE and less accurate 95% simulation intervals, even within proportional hazards data-generating scenario.

Comparison goodness of fit tests (Hosmer-Lemeshow test and AIC criterion) revealed that, under a different range of data conditions, Gamma and Weibull regression models were better behaved. Finally, investigation of the pattern of the residuals as a function of X, which have been implemented by the mean of the residuals across deciles of X, showed more bias for the Cox proportional hazard model across all generated data and sample sizes (see Figures 2-15).

## Discussion

Although there are many substantial studies addressing the statistical issues in healthcare cost analysis over the last few decades, it is still an important issue that needs further evaluation. In this paper, we assessed the performance of various well-known statistical regression-based models in healthcare expenditure analysis, through different sample sizes and data-generating processes, using a Monte Carlo simulation. Each model was evaluated on 10,000 batch random samples, with 25, 50, 100, 500 and 1,000 sample sizes. Other studies were performed using just one large sample size (such as 10,000) [5,10], while we know the sample size is an important issue in healthcare studies and in precision of model-based estimators.

We found that, by considering the different interest points of various research and various data conditions, different model-based estimators could be used. Indeed, no estimator is considered to be the best across all ranges of data-generating processes. In addition, the accuracy of the results was almost the same in all sample sizes.

However, the GLMs estimated population means more precisely in all data-generating processes and sample sizes. In this respect, our results are consistent with other studies [2,5,6,10]. Comparative studies between log models were evaluated on 1,000 random samples, with a sample size of 10,000. They found almost identical results in estimating the slope β_1_, but the GLMs were substantially more precise than OLS-based model [5]. In this paper, as the sample size increased, the precision of estimating the mean population and the β_1_ using an OLS-based model became closer to that of GLMs.

Based on our result, the Gamma regression model provided more accurate estimates of population mean. In other studies, which compare log and Cox proportional hazard models, the Gamma regression model was introduced as the reasonable model across all of the simulation processes [13]. They have also found that the Cox proportional hazard model exhibited good performance when data were generated by distribution with a proportional hazards assumption [13]. In this paper, a Weibull distribution was selected as the proportional hazard data-generating mechanism. In addition, investigating proportional hazards assumption detected that gamma generation process also has produced data with proportional hazard properties but the Cox proportional hazard model showed a poor result within these data generation process. We also found that the Cox proportional hazard model behaved poorly in other data generation scenarios.

Our study has some limitations, including the fact that our focus was on generating skewed data, while kurtosis may have affected the results. Furthermore, the study was limited to fixed covariates.

## Conclusions

Selecting the best model is dependent on the interest point of research, which could be the estimated mean of the population or covariate effects. There is no best model among all data conditions. It seems that the GLMs, especially the Gamma regression model, behave well regarding the estimation of population means of healthcare costs in most of the conditions. The results are consistent among all sample sizes; however, increasing sample size leads to improvement in the performance of the OLS-based model.

Based on estimation of the β_1_, GLMs seems to provide plausible estimations and as the sample size increased, estimated the β1 more precisely in all data-generating processes. Under all data generation, process even proportional hazard data generation scenarios the Cox proportional hazard model provided a poor estimation of mean population and the β_1_.

## Abbreviations

Prob. H.L.: Hosmer–Lemeshow test
signif.: At the 5% level
